# P-1643. Cardiovascular Manifestations of Persistent COVID in Mexico

**DOI:** 10.1093/ofid/ofaf695.1819

**Published:** 2026-01-11

**Authors:** Luis Del Carpio-Orantes, Daniela Trelles-Hernández, Sergio García-Méndez, Mayra Evelyn Quiñones-Martínez

**Affiliations:** Grupo de Estudio para el Diagnóstico y Tratamiento de COVID-19 en Veracruz, México, Veracruz, Veracruz-Llave, Mexico; Grupo de Estudio para el Diagnóstico y Tratamiento de COVID-19, Veracruz, Veracruz-Llave, Mexico; Grupo de Estudio para el Diagnóstico y Tratamiento de COVID-19, Veracruz, Veracruz-Llave, Mexico; Hospital de Alta Especialidad de Veracruz, Veracruz, Veracruz-Llave, Mexico

## Abstract

**Background:**

Persistent COVID refers to the persistence of COVID-19 symptoms 12 weeks after the acute phase. In some geographic regions, such as Europe, there is a high prevalence of cardiovascular manifestations, including various dysautonomias, in addition to other persistent manifestations. The objective of this study is to identify the main cardiovascular manifestations of persistent COVID in Mexico.

**Methods:**

An adult population of patients who meet the criteria for persistent COVID based on symptoms and a PASC score greater than 12 points, which establishes the clinical diagnosis of persistent COVID, was analyzed.

**Results:**

203 subjects were analyzed; 68% were female, with a mean age of 41.8 ± 11.3 years. 66.0% of the subjects presented two or more episodes of SARS-CoV-2 infection, 29.6% presented severe disease, and 70.4% presented mild to moderate disease. 89.7% were vaccinated against COVID-19: 6.9% with one dose; 31.5% with two doses; and 51.2% with three or more. Cardiovascular symptoms were reported in 82.8% of patients: 56.2% with palpitations, 43.3% with tachycardia, 27.1% with chest pain, 18.7% with hypertension, 14.8% with hypotension, 11.8% with edema, 9.9% with arrhythmias, 5.9% with syncope, 4.9% with myocarditis, and one subject (0.5%) with a heart attack. Three cases of dysautonomia type I were reported in this patient group. Cardiovascular symptoms were more persistent in women than in men (87.0% vs 73.8%, p = 0.028); Among these, palpitations (61.6% vs. 44.6%, p = 0.033), edema (15.4% vs. 4.6%, p = 0.029), and tachycardia (47.8% vs. 33.8%, p = 0.069) were more frequently identified in women.

Risk associations for cardiovascular symptoms were identified with the following variables: male sex, having had COVID-19 two or more times, having had severe COVID-19, persistence of pulmonary symptoms, persistence of gastrointestinal symptoms, and persistence of musculoskeletal symptoms.

**Conclusion:**

Cardiovascular manifestations of persistent COVID have a high prevalence in the Mexican population.

**Disclosures:**

All Authors: No reported disclosures

